# Quercetin: A Bioactive Compound Imparting Cardiovascular and Neuroprotective Benefits: Scope for Exploring Fresh Produce, Their Wastes, and By-Products

**DOI:** 10.3390/biology10070586

**Published:** 2021-06-26

**Authors:** Irshad Ul Haq Bhat, Rajeev Bhat

**Affiliations:** ERA-Chair for Food (By-) Products Valorisation Technologies (VALORTECH), Estonian University of Life Sciences, 51006 Tartu, Estonia; rajeev.bhat@emu.ee

**Keywords:** quercetin, secondary metabolite, cardioprotective and neuroprotective agent, fruits and vegetable wastes, by-products, circular economy

## Abstract

**Simple Summary:**

The present review summarizes the generated hypothesis and critical role of quercetin in cardiovascular and neuroprotective diseases reported over the last decade. In this review, 335 research articles were referred from the popular scientific database (e.g., Scopus and Web of Science) to elaborate on the importance of quercetin in addressing human ailments and the health-protective role imparted. Being a valuable bioactive compound within the circular economy context, the source for obtaining quercetin has been extended for food industrial wastes/byproducts (mainly of fresh produce). Further, the establishment of the molecular mechanism, antioxidant potential, oxidative stress, metabolic process, myocardial damage, anti neurogenerative potential, enzymatic expression, and ROS inhibition renders quercetin an ideal phytochemical that can provide protective benefits against cardiac and neurodegenerative diseases.

**Abstract:**

Quercetin, a bioactive secondary metabolite, holds incredible importance in terms of bioactivities, which has been proved by in vivo and in vitro studies. The treatment of cardiovascular and neurological diseases by quercetin has been extensively investigated over the past decade. Quercetin is present naturally in appreciable amounts in fresh produce (fruits and vegetables). However, today, corresponding to the growing population and global demand for fresh fruits and vegetables, a paradigm shift and focus is laid towards exploring industrial food wastes and/or byproducts as a new resource to obtain bioactive compounds such as quercetin. Based on the available research reports over the last decade, quercetin has been suggested as a reliable therapeutic candidate for either treating or alleviating health issues, mainly those of cardiovascular and neurological diseases. In the present review, we have summarized some of the critical findings and hypotheses of quercetin from the available databases foreseeing its future use as a potential therapeutic agent to treat cardiovascular and neurological diseases. It is anticipated that this review will be a potential reference material for future research activities to be undertaken on quercetin obtained from fresh produce as well as their respective processing wastes/byproducts that rely on the circular concept.

## 1. Introduction

**Figure 1:**
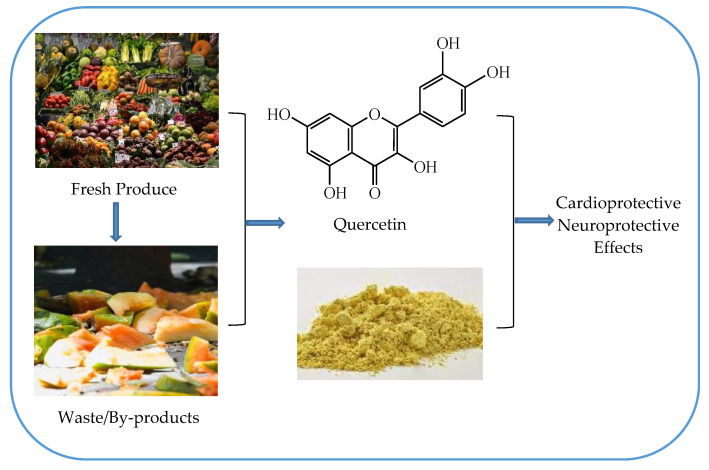
Quercetin: Sources, structure and uses.

Quercetin is a carbohydrate-free flavonoid, a secondary metabolite found in flora, including fresh produce (fruits and vegetables), some of which include red onion, apples, and berries. These compounds have been studied to evaluate the biological activities of flavonoids [1]. The overview depicting the theme of this review is given in Figure 1 [2,3,4]. These properties of quercetin have been attributed primarily to their antioxidant capacity and free radical scavenging ability. Quercetin intake has been found to affect mitochondrial biogenesis, energy production, electron transport chain performance, ROS production modification, and mitochondrial defect modification [5,6]. The antioxidant potential and free radical scavenging ability have played a major role in explaining quercetin’s neuroprotective effects with the exceptional property of passing the blood–brain barrier. Thus, based on the thorough literature analysis (past decade) from the available databases (in Scopus and Web of Sciences), this review is being designed for cardioprotective and neuroprotective effects. However, to the authors’ knowledge, there have been very limited reviews published that have shown cardioprotective and neuroprotective properties for quercetin.

On another note, currently, the food industry’s post-processing wastes and byproducts have gained much importance in the present-day circular Bioeconomy concept scenario. In the circular economy, an approach contrary to the traditional linear economy (TLE) is adopted; in TLE, products after use are thrown away. These products are manufactured by the extraction and processing of raw materials, usually non-renewable resources. However, the Bioeconomy (BE) includes the production of renewable biological resources and the transformation of these resources and waste streams into added-value products. The meeting point of these three economic concepts as an intersection is the Circular Bioeconomy Concept (CBEC), where the raw material, e.g., biomass, is utilized as renewable resources as it includes byproducts, residues, and waste. This approach retains raw material in a closed material loop and hence enhances productivity. In addition, raw material by the process of recycling can be converted into prototypes of different uses. Fruits and vegetable wastes/byproducts, being a cheap source of raw materials, have been recommended to be exploited for the isolation of bioactive and other value-added compounds. Besides, these wastes and byproducts also help towards minimizing environmental pollution-based issues [7,8,9,10]. Hence, there is ample scope to obtain bioactive compounds such as quercetin from fruit and vegetable-based industrial wastes and byproducts. With this background, this review aims to provide guidance and reference for future cardioprotective and neuroprotective aspects of quercetin. Besides, additional focus is also laid towards identifying the potentiality of exploiting food industrial wastes and byproducts to obtain quercetin

## 2. Discussion

### 2.1. Quercetin as a Cardioprotective Agent

Based on pharmacological evidence, quercetin obtained from fruits and vegetables has the tremendous ability to act as a cardioprotective agent. The muscular function is greatly influenced by its antioxidative and antiplatelet properties. These properties prompt actions such as anti-smooth muscle cell proliferation and migration, which in turn improves cardiac cell mitochondrial function and inhibits nuclear factor-kappa light chain-enhancer of activated B cells (NF-κB), as stated by Ay et al. [11]. Sengul et al. [3] reported protective effects of quercetin in rats and demonstrated that the dosage of 50 mg/kg and 100 mg/kg of quercetin have significant protective effects on the 5-FU-induced cardiotoxicity in rats after 14 days of administration. Quercetin can reduce cardiovascular disease linked to brain-derived neurotrophic factor (BDNF); in this aspect, Wang et al. [2] stated that quercetin exerted significant antidepressant and cardioprotective effects in mice. A diet of high fat is linked to perturbations in the metabolism of fatty acids and contributes to cardiac diseases such as cardiometabolic syndrome and cardiac damage, which can be alleviated by quercetin by restoring myocardial microcirculation as studied by Yu et al. [12]. The beneficial effects of quercetin help in decreasing plasma triglycerides and intramyocardial fat deposition [12]. Rolnik et al. [13] investigated quercetin derivatives for the development of new anti-platelet strategies and management of current therapies in cardiovascular diseases linked to hyper-activation of platelets. Further, Elbarbry et al. [14] have elaborated the role of quercetin by studying its role in lowering blood pressure. The effect of quercetin in spontaneously hypertensive rats (SHR) was studied by administrating different concentrations of quercetin. The enzymatic activity of rats suggested that medium and high doses of quercetin resisted high blood pressure. They hypothesized that quercetin has anti-hypertensive and provided a novel mechanism for its underlying cardioprotective properties [14]. The protective effects of quercetin against isoproterenol-induced myocardial ischemia and verification of the cellular mechanisms based on the L-type Ca^2+^ channel (LTCC), Ca^2+^ transients, and myocardial contractility was reported by Liang et al. [15]. In their animal model, they hypothesized that quercetin gives a cardioprotective effect by inhibiting Ca^2+^ influx and contractility in experimental animals. Besides, the ‘Zi Shen Huo Luo Formula’ (ZSHLF), a traditional Chinese-medicine-(TCM)-based herbal formula, consists of many drug material components. Quercetin is among these components that tend to exhibit cardioprotective effects, as studied by Song et al. [16]. They supported their results by establishing a mechanism by carrying in vitro studies using rats. However, they also insisted that in ZSHLF, major bioactive components or metabolites are responsible for the effects and the implications of these findings in patients and further research is needed. The inhibition of myocardial damage due to recurring chest pain is the main issue in heart disease. The results indicated that the cardioprotective effect of QPC highlights the importance of anti-arrhythmogenic during ischemic heart disease. These results can be a milestone for a new direction for the treatment of myocardial infarction. Quercetin, along with its derivatives, such as rhamnetin, rutin, and hyperoside, has been reported to impart a positive influence on the cardiovascular system [17]. Further, Ferenczyova et al. studied the effects of quercetin and its metabolites on cardiac injury through their antioxidant, anti-inflammatory, and molecular pathways-modulating properties. However, it was revealed that due to a low number of clinical trials focused on cardiac effects of quercetin and its derivatives, clinical data were inconclusive [18]. In their other study, cardiovascular benefits of quercetin in diabetic rats of different ages. It was reported that quercetin might be advantageous for vascular function in diabetes type 2; however, increasing age and/or progression of diabetes may confound its vasculoprotective effects. Though, quercetin was not so effective in preventing myocardial ischemia-reperfusion (I/R) injury in type 2 diabetes and older age [19]. Wang et al. [20], after establishing the structure of quercetin by spectroscopic data, studied its cardioprotective properties. The cardioprotective effects of quercetin against oxidative stress of H9c2 cells induced by H_2_O_2_ showed quercetin derivatives exhibited protective effects against H9c2 cells injury induced by H_2_O_2_ The cardioprotective effects of quercetin-containing drugs, corvitin and lipoflavon, were reported first time by Stechyshyn and Pavliuk [21]. Quercetin can relieve endoplasmic reticulum dysfunction in Pak2-CKO hearts. The findings of using quercetin uncovered a new cardioprotective mechanism and therapeutic strategy for encouraging options for treating cardiac disease and heart failure [22]. The cardioprotective index (CI) has been studied in diabetic rats after treatment with quercetin for 28 days. Ogar et al. [23] attributed the enhanced CI to the use of quercetin. The cardioprotective effects of quercetin, along with a dietary supplement, alpha-linolenic acid, have been described by Burak et al. [24].The quercetin derivative was used to study cardiac dysfunction, scavenging free radicals, and reduced lipid peroxidation, as reported by Shu et al. [25] and attributed cardioprotective effects of quercetin derivative to partially counteracted PI3K/Akt inhibitor LY294002. Wang et al. [26] isolated glycosidic derivative quercetin and studied their cardioprotective effects against H_2_O_2_-induced apoptosis in H9c2 cells. At micromolar concentrations, the quercetin glycosides exhibited stronger activity than the acylated quercetin glycosides.

Chakraborty et al. [27] studied quercetin in combination with curcumin as a bio enhancer for ischemia–reperfusion injury (IRI) induced myocardial toxicity in rats. After 30 days of treatment, it was observed that a combination of these two phytoconstituents exhibited intense protection of IRI-induced myocardial toxicity and was further supported by pharmacodynamic interaction [27]. The cardioprotective effects of quercetin in animals exposed to intermittent hypobaric hypoxia (IHH) were studied by Chis et al. [28]. The oxidative/nitrosative stress increases by hypobaric hypoxia in heart tissue and quercetin act as a cardioprotective agent, which reduces the oxidative cardiac dysfunction induced by oxidative/nitrosative stress [28]. Quercetin administration in a group of rats through myocardial energy metabolism restoration gives a prophylactic effect in response to adriamycin-induced toxic effects [29]. LC-MS/MS analysis was performed for the detection and determination of the bioactive constituents, including quercetin, in methanol extract of *Alternanthera philoxeroides* (Mart.) Griseb. It was revealed by in vitro cell evaluation studies that methanol extracts significantly stopped cardiomyocyte apoptosis [30]. The cardioprotective effects of quercetin against the damage induced by a high-cholesterol (HC) diet in hyperglycemic rats was studied, which was attributed to intracellular antioxidant mechanisms and bioenergetics [31]. These researchers observed that quercetin reduced HC-induced changes in the lipid profile and glycemia in experimental rats. The systolic cardiac function was enhanced in mice administrated with quercetin. The rapid metabolization of quercetin to tamarixetin was maintained at a higher concentration for enhancement of systolic cardiac function, as reported by Hayamizu et al. [32]. Quercetin can exert cardiotonic action via digitalis-like enhancement of Ca^2+^ transients by itself and its metabolite, tamarixetin.

The effects of quercetin on cardiac parameters and ischaemia–reperfusion (I/R) injury, the Langendroff method was used on male Wistar rats by Garjani et al. [33]. The reported results indicated that at lower quercetin concentrations, a protective effect against I/R injuries in isolated rat hearts was observed. The conjugation of quercetin with the anticancer drug doxorubicin was evaluated by Alrushaid et al. [34] to understand its cardioprotective effects and the mechanism involved. The cardioprotective mechanism of conjugated doxorubicin–quercetin involved scavenging of ROS and CYP1B1 inhibition. The cardioprotective roles of quercetin against I/R injury and the underlying mechanism based on molecular signaling were reported by Li et al. [35]. The model based on oxygen-glucose deprivation/reoxygenation (OGD/R) of I/R was reported in myocardial H9c2 cells in the absence or presence of quercetin. Their results supported the fact that a dual character of quercetin is involved in the process of myocardial I/R. The extensive data reported by Ballmann et al. [36] revealed that quercetin improves various metabolic processes such as mitochondrial biogenesis and antioxidant enzymatic action. The availability of quercetin facilitates dystrophin-associated glycoprotein complex (DGC) assembly and decreases inflammation in dystrophic hearts. Along with other polyphenols, quercetin was studied for cardioprotective potential in different fractions of *Syzygium cumini* L. seeds [37]. The control of angiotensin-converting enzyme (ACE), HMG-CoA reductase, LDL oxidation, and tertiary butyl hydrogen peroxide (TBHP) was exhibited by *Syzygium cumini* L. seed fractions rich in quercetin content. The investigation on streptozotocin-nicotinamide-induced adult male diabetic rats administrated quercetin with different concentrations orally for 28 days. A decrease in glucose levels in addition to the reduction in other necessary parameters, including cardiac injury marker levels, were found. They hypothesized that quercetin could be used to improve myocardial damage due to oxidative stress, inflammation, and apoptosis in diabetes [38]. In preventive therapy for cardiac disease, cardioprotection represents one of the most important and realistic aspects, and quercetin helps to a great extent in providing cardioprotection. Kumar et al. [39] explored the cardioprotective activity of quercetin and related mechanisms. Wister rats were given quercetin orally for 14 days. It was observed that quercetin pre-treatment exhibited protective effects on the heart. Isoproterenol-induced oxidative stress, inflammation, protection of heart architecture, and down-regulation of the expression of calpain were significantly reduced by quercetin treatment. Thereby concluded that quercetin has cardio-protective potential and established its mechanism of action against isoproterenol-induced myocardial infarction (MI) in experimental rats. The inhibition of chronic-unpredictable-stress-(CUS)-induced left ventricular dysfunction (LVD) in rats by quercetin in an animal model was studied by Bin-Jaliah [40]. It was hypothesized that quercetin tends to prevent myocardial infarction (MI) induction by CUS. The rats were treated with a constant dosage of quercetin for 3 weeks. The measurable parameters, such as blood pressure and electrocardiogram (ECG), along with histological study, were taken into consideration prior to quercetin treatment. It was observed that quercetin treatment lowered blood pressure and substantially prevented MI attributed to the blocked elevation of the ST-segment on the ECG. Furthermore, the significant inhibition of expression of Bax RNA messages and blocked CUS-induced TNF-α and IL-6 upregulation led to the efficacy of quercetin treatment. Thus, a possible therapeutic role for quercetin in CUS-induced cardiac dysfunction was established by Bin-Jaliah [40]. Quercetin was studied for its effect on myocardial ischemia in patients with stable coronary heart disease (CHD) by Chekalina et al. [41]. With the intake of constant dosage of quercetin for 45 days, the deceleration time (DT) value and premature ventricular contraction (PVC) number dropped significantly by the usage of quercetin. Thus, the cardioprotective properties of quercetin in conditions of CHD were recognized. The generation and excessive production of reactive oxygen species (ROS) lead to oxidative stress in the body and are considered major reasons for cardiovascular diseases (CVDs). The production of reactive radicals is one of the major management strategies for CVDs along with other factors and can be controlled by quercetin, as reported by Ramachandran et al. [42]. Quercetin treatment for isoproterenol-induced toxicity in H9c2 cardiomyoblast cells was studied. The treatment with quercetin prompted a protective effect by augmenting the antioxidant prominence of the cells [42]. Thus, based on animal models, oxidative stress, metabolic process, myocardial damage, enzymatic expression, ROS inhibition, and cell line studies, an extensive approach has been opted to establish the importance of quercetin as a cardioprotective agent. Some of the selected works reported on the role of quercetin as a cardioprotective agent are given in Table 1.

### 2.2. Quercetin as a Neuroprotective Agent

A unique four-step strategy comprising of ‘Global profiling’, ‘Chemical structural classification’, ‘Intra-group screening’, and ‘Component-knockout optimization’ was adopted to screen and optimize the functional compound combination (FCC) from traditional Chinese medicine (TCM). The holistic neuroprotective effects of the FCC consisting of quercetin indicated antioxidative and anti-inflammatory responses in PC12 cells to save them from oxidative stress, as reported by Wang et al. [71]. The group hypothesized that the reported strategy could offer an alternative to discover phytochemicals of biological importance, such as quercetin used in traditional medicines. The hexane extract of *Spondias mombin* L. (Anacardiaceae) ‘Cajazeira’ containing quercetin has shown a neuroprotective effect via anxiolytic and antioxidant activities in zebrafish [72]. The study on elaboration mechanism explaining the effects of extract containing quercetin on neuroprotective benefits was established. *Eclipta alba* L. ‘Bhringra’ hydroalcoholic extract containing quercetin along with other phytoconstituents was reported by Bhatia et al. [73]. It was further stated that cellular antioxidant and enzymatic expression showed improvement by using *Eclipta alba* L. extract, therefore projecting this as a potential plant for the generation of the therapeutically neuroprotective agent. The first-time isolation and identification of quercetin glycosidic derivative from *Hibiscus rosasinensis* L. was reported by Shen et al. [74]. This derivative was suggested for the prevention of Alzheimer’s disease. The effect of quercetin-loaded microcapsules was analyzed for neuroprotective properties in Wistar adult rats by studying nitrergic neurons reducing oxidative damage [75]. In a comparative study by Jalili-Baleh et al. [76], quercetin was chosen as the standard for a neuroprotective activity for chromone–lipoic acid hybrids. The use of quercetin can protect retinal cells from deterioration and had not dilapidated their function [77]. The researchers related the protective effect of quercetin and other phytoconstituents to higher expression of certain proteins (photoreceptor-specific proteins), photoreceptors (rhodopsin and cone opsins), and at the same time to reduced expression of particular inflammatory markers.

The dietary intake of quercetin was recently studied in an animal model (mice) by Zhang et al. [78] to establish its role in reducing depression, a true neurological problem. These findings supported astrocyte reactivation in the regulation of quercetin neuroprotection and hypothesized that a quercetin-rich diet intake in fruits, vegetables, and food additives would relieve the stress. The effect of injected quercetin at different intervals in neonatal mice with hypoxic–ischemic brain injury was reported by Le et al. [79] to study its neurological functions. The administrated quercetin protected microglial cells (BV2 cells) from oxygen-glucose deprivation (OGD) induced damage; further microglial oxidative stress-related molecules were intercepted. The hepato-neuroprotective effect of extracts of *Barnebydendron riedelii* Tul. was attributed to its quercetin content by Baraka et al. [80]. It was stated that glycosidic quercetin, through its modulatory effects on NF-κB/IL-6 and Nrf2/HO-1 signaling pathways, is enabled to exhibit a protective effect. A quercetin-integrated Cyclodextrin complex was studied for its neuroprotective behavior in nose-to-brain Que’s delivery systems [81]. The obtained results were warned further ex vivo and in vivo studies for delivery of specific targeted delivery. Quercetin and quercetin 4′-*O*-glucoside were biologically evaluated for cerebral ischemia-reperfusion in mice. The quercetin extractives from different extracts showed different neuroprotective effects. It was suggested that further evaluation for neurotoxic effects and appropriate neuroprotective mechanisms should be continued to explore [82]. The neurotoxic damage induced by Bisphenol-A (BPA) in zebrafish (*Danio rerio*) and the effect of quercetin supplement treatment was carried out by Sahoo et al. [83]. The neurobehavioral response, oxidative stress, and neuromorphological changes were observed by the use of quercetin and it was considered as an effective agent to intervene in the induced neurotoxicity in Zebrafish by BPA. The delivery of drugs while crossing the blood–brain barrier (BBB) to a selected site to treat Parkinson’s disease (PD) is a challenging issue. Thus, modification in treatment is needed; in this aspect, nanoparticles were used to carry the drugs to the selected target. Quercetin is an anti-Parkinson disease agent, and recently Liu et al. [84] used gold nanoparticles loaded with quercetin to study the enhancement in BBB permeability of quercetin via the photothermal effect. The results revealed that by this treatment, quercetin was accumulated in the brain and it was further hypothesized that nanoparticles could have the potential for neuroprotective drug delivery and treatment for PD. In another study, quercetin nanoparticles were studied for their better availability. A constant dose of quercetin nanoparticles was administrated to animals along with AlCl_3_. Based on microscopic results, nanoparticles showed neurogenerative changes and improvement of tyrosine hydroxylase (TH), and this study was the first study revealing the therapeutic effects of quercetin nanoparticles for Alzheimer’s disease (AD) model and further investigation was warned by Rifaai et al. [85]. Neuroprotection against cerebral ischemia/reperfusion injury in rats was studied by Wang et al. [86] using quercetin oral pretreatment. Neuroprotective parameters such as brain infarction, blood–brain barrier disruption, oxidative stress, TNF-α, and IL-1β mRNA expression, along with apoptotic caspase 3 activity, and with neuroprotective, anti-oxidative, anti-inflammatory, and anti-apoptotic effects of quercetin, were reported. The increase in ERK/Akt phosphorylation and protein phosphatase activities and at the same time inhibition of ERK or Akt were studied to report the cause of apoptotic cell death and cytotoxicity in hippocampal slice cultures and neuron/glia cultures. In conclusion, it was hypothesized that the role of quercetin was prominent in improving the protein tyrosine and serine/threonine phosphatase activity, followed by reduction of ERK and Akt phosphorylation, thereby addressing the important role of quercetin in understanding its neuroprotective behavior [86]. The administration of quercetin supplementation leads to an increase in the bioavailability of NO in the jejunum in euglycemic diabetic rats. This treatment reduces the effects of diabetes on nNOS-IR neurons and VIP-IR varicosities in the myenteric plexus of experimental diabetic rats, as reported by Martins-Perles et al. [87]. The presence of quercetin in *Camellia nitidissima* Chi, ‘Tea for Longevity’ a well-known plant consumed as both medicine and food in China, was studied for its neuroprotective effect by An et al. [88]. Based on two used terms, ‘synergistically boosting endogenous antioxidant defences’ and ‘neurotrophic signalling pathway’ by An et al. [88], this plant has been cited as an important source for phytonutrients, including quercetin for edible foods and beverages. Soxhlet extraction to yield quercetin along with other phytoconstituents from *Pinus roxburghii* Sarg. by Sharma et al. [89] for in vivo and in vitro studies was carried out to evaluate neuroprotective activity in rats. It was reported by Karaman et al. for the first time that quercetin obtained from mushrooms (*Coprinus comatus* (O.F.Mull.) Pers.) exhibited neuroprotective activity [90]. This plant has been cultivated in China and can be used as an acetylcholinesterase (AChE) inhibitory agent. The work on dopaminergic human SH-SY5Y cell lines using quercetin was evaluated for the neuroprotective role [91]. The impact of rotenone-induced neurotoxicity in SH-SY5Y cell lines was studied by correlating intracellular reactive oxygen species (ROS) and imbalance in the mitochondrial membrane potential (MMP). It was established that quercetin gave cells protection against rotenone; this quercetin was hypothesized as a therapeutic agent with significant neuroprotective potential. The neuroprotective effect and control of gut microbiota linked with nerve damage in diabetic rats using quercetin were studied by Xie et al. [92]. It was suggested that quercetin has a therapeutical potential to reduce diabetic peripheral neuropathy (DPN) in rats and can be considered as a key component for the treatment of such neuropathic-related issues. The role of quercetin in treating neurotoxicity caused by metronidazole by obstructing the proportion of antioxidants in the brain tissue and inducing nitric oxide synthesis along with apoptosis. Quercetin was found active to protect against such neural damage by Chaturvedi et al. [93]. Nanoparticles can enhance the delivery of quercetin, as stated by Pinheiro et al. [94]. The blood–brain barrier was targeted by such designed nanoparticles. By this approach, the neuron was protected against amyloid-beta fibrillation; thus a targeted delivery of quercetin was achieved, and a novel strategy to treat Alzheimer’s disease can be generated. The hydroalcoholic extract of coffee silverskin containing quercetin along with other polyflavonoids was reported for its neurodegenerative activity attributed to counteract oxidative stress and maintain cell viability [95]. The neuroprotective activities in rats using quercetin were determined by de Mattos et al. [96]. The neuroprotective properties of quercetin for treating autism in an animal model is being hypothesized as an important therapeutic candidate for addressing neurological disorders such as autism spectrum disorders (ASDs). Quercetin with hydroxyl groups on the ortho position can have been reported to provide enhanced neuroprotection as reported by Lossi et al. [97]. This study contributed towards a new finding in the translation application of polyphenols, as stated by the researchers. The spectroscopic analysis was used to characterize lipid nanoparticles encapsulated with quercetin and used as neuroprotective agents. The results revealed that the nanoparticles are appropriate for brain applications, particularly for Alzheimer’s disease, due to the inhibition of amyloid-beta aggregation [98]. Quercetin was found as a major constituent in the methanolic stem extract of *Colebrookea oppositifolia* Smith. with a potential of cerebroprotective activity against ischemia–reperfusion-induced brain injury during in vivo studies [99]. The oral administration of quercetin at constant dosage in mice was studied for the counter effects of Aflatoxin B1, a mycotoxin in mice. The antioxidant effect of quercetin led to a preventive role against oxidative stress by supporting antioxidative defense systems and restraining the lipid peroxidation process [100]. *Euonymus alatus* (Thunb.) Siebold. a food plant consumed as traditional medicine in East Asian countries, has been found rich in quercetin. The extract from the plant was utilized for anti-neurotrophic factors. The defense in the mouse hippocampus from scopolamine-induced damage through as brain-derived neurotrophic factor-mediated Nrf2 activation was established by the use of quercetin by diminishing cognitive decline [101]. The potential toxin in diet responsible for the pathogenesis of Alzheimer’s disease (AD) is dietary advanced glycation end products (dAGEs), as stated by Yang et al. [102]. Thus, experimental mice were treated with quercetin after giving dAGEs for 21 days. The results evaluating different biochemical pathways suggested that treatment by quercetin supplication has improved cognitive function in experimental animals. A similar study was carried out by Yang et al. [103] by stating that extended application of quercetin may be beneficial for high consumers of dAGEs. The neurotoxicity by the exposure of pesticide fenitrothion has led Ibrahim et al. [104], to carry out a study in rats for the role of quercetin against the development of such neurological impairment. The study leads to the hypothesis that quercetin can prove helpful against fenitrothion by downregulating apoptosis-related genes and catecholamines. Thus, it can be exploited as a neuroprotective agent. Shalavadi et al. [105] carried out the neuroprotective capability of *Convolvulus pluricaulis* Choisy extracts and their comparison with quercetin in mice. Diabetic encephalopathy (DE) can be improved by the use of quercetin as established by studying the SIRT1/NLRP3 pathway by Vieira-Frez et al. [106]. The crucial link between the use of quercetin and neuroprotective efficacy in diabetic encephalopathy (DE) was reported by them in mice. A similar study was reported by Hu et al. [107] wherein SIRT1/NLRP3 pathway was considered a crucial mechanism for the neuroprotective effect against DE using quercetin in mice used as a diabetic model animal [107]. The results presented by Park et al. [108] were able to justify that quercetin can be considered as an active component for mediating the neuroprotective role by controlling thioredoxin expression and maintaining the interaction between apoptosis signal-regulating kinase 1 (ASK1) and thioredoxin, an antioxidant. The findings suggest that quercetin mediates its neuroprotective function by regulation of thioredoxin expression and maintenance of interaction between ASK1 and thioredoxin. Quercetin in combination with piperine was studied for neuroprotective effect against rotenone- and iron-supplement-induced PD in experimental rats. It was found that this combination was quite effective in treating Parkinson’s disease (PD) in rats [109]. The neuroprotective effects of quercetin in Wistar rats as subarachnoid hemorrhage (SAH) model animals for its role in visualizing the effects against cerebral vasospasm was reported by Gül et al. [110]. The biochemical and histopathological evaluation reported revealed that quercetin has a crucial role in SAH therapy by stopping vasospasm. The copper-induced neurodegeneracy in injured P19 neuronal cells can be countered by the use of quercetin, as stated by Zubčić et al. [111]. MTT assay, preventing ROS formation, caspase-3 activation, and chromatin condensation by using 150 μM quercetin was the basis to prove the neuroprotection offered by it. However, further detailed pharmacological and toxicological studies to establish its role were warned. The controlled and sustained release of quercetin at targeted neuron cells was carried out by loading quercetin poly (D,L-lactide-co-glycolide)-poly(ethylene-glycol)-poly(D,L-lactide-co-glycolide) (PLGAPEG-PLGA) hydrogel. The results obtained suggested that oxidative damage and inflammation in the spinal cord were improved along with increase neuron survival rate and promotion in nerve regeneration and motor function recovery. The authors suggest that the sustainable approach of quercetin delivery has promising applications in the clinical treatment of brachial plexus avulsion (BPA) [112]. Based on the study carried out by Yu et al. [113] the increase in the survival rate of PC12 injured by Aβ25-35, promotion of cell proliferation, and antagonization of the toxicity of Aβ due to quercetin, led it to be considered as a strong agent as a drug candidate for the treatment of Alzheimer disease. The neurotoxic effects of styrene 7,8-oxide (SO) and its cytotoxicity mechanism were studied using quercetin as a neuroprotective agent. The presented results were in agreement that cytotoxicity of SO was mediated by oxidative stress and apoptosis, necrosis, and necroptosis mechanisms, and at the same time, quercetin delivered the neuroprotection [114]. Quercetin 3, 7-*O*-α-L-dirhamnoside, a quercetin derivative, was found actively in providing a protective effect against induced neurotoxicity [115]. The brain targeting of quercetin-loaded exosomes enhanced its bioavailability in Alzheimer’s disease (AD) and further relieved the symptoms of AD via inhibition of biochemical pathways such as cyclin-dependent kinase 5 (CDK5)-mediated phosphorylation of phosphoprotein Tau and decrease of the formation of insoluble neurofibrillary tangles (NFTs) [116]. Malik et al. [117] and Gao et al. [118] exploited extracts *Lactuca sativa* L. and *Lycium ruthenicum* Murr. extracts rich in quercetin content, and proved their neuroprotective potential. Chronic neuroinflammation is the leading cause of anxiety issues. Quercetin has been proven to reduce such inflammation and can be considered as a potential agent for inhibiting anxiety-like symptoms in neuropsychiatric diseases, as stated by Lee et al. [119]. Nanoquercetin via endocytic cell proliferation can be slowly released as seen in the model studies and has shown better action as compared to quercetin, thus can be considered as a new milestone for providing treatment for Alzheimer’s disease [120]. The Enteric Nervous System (ENS) is affected by arthritis, and Piovezana Bossolani et al. [121] studied the impact of quercetin alone and in combination with ibuprofen for 60 days in rats and found that combination treatment was found to be remarkably effective in treating arthritis, thus ENS [121]. Quercetin derivative was also found to be effective in diabetic rats to control neurological damage [122]. Quercetin exhibited neuroprotective effects on cell viability, morphology, and gene expression following corticosterone exposure, as reported by Donoso et al. [123]. It was hypothesized that quercetin defends cortical cells against corticosterone-induced cytotoxicity and, at the same time, improves cell survival through the Nrf2 pathway and expression of Fkbp5. Similarly, Nrf2 dependent HO-1 signaling by quercetin derivative was found a potential agent in addressing Parkinson′s disease management [124]. Quercetin was found to bind regulatory sites in α7-PAM, leading to a reduction in reactive oxygen species. This allosteric property of quercetin, as reported by Nielsen et al. [125] was considered as scaffolds for the development of new neuroprotective therapeutic candidates. The calorimetric MTT assay was carried out to investigate the effect of quercetin as compared with estrogen in its role in Alzheimer’s diseases and was able to establish its role in neuroprotection [126]. The blueberry extracts rich in quercetin were administrated in mice at different concentrations. The neuroprotective and antidepressant-like effects were attributed to the presence of a high concentration of flavonols and pigments such as quercetin and anthocyanin, respectively [127]. Similarly, *Viola cornuta* L. and *Viola* x *wittrockiana* Gams edible flower extracts were examined for neuroprotective effect against paralysis in round worm (*Caenorhabditis elegans)* by Moliner et al. [128]. They claimed that quercetin derivative quercetin-3-*O*-(6-*O*-rhamnosylglucoside)-7-*O*-rhamnoside was responsible for the neuroprotective properties. *Pistacia lentiscus* L. leaves extract (PL) containing quercetin and quercetin rhamnoside also exhibited neuroprotective potential investigated in a mice animal model, as mentioned by Azib et al. [129]. The synergetic effect of quercetin in combination with sitagliptin has shown neuroprotective potential in β-amyloid (Aβ)-induced Alzheimer’s disease in male Sprague Dawley rats as reported by Li et al. [130]. Wu et al. [131] treated brain-injured rats with quercetin and hypothesized that quercetin could be used as a therapeutic drug to alleviate the injury caused in the brain by hypoxic ischemia. Quercetin, in its neuroprotective role, was considered to prove a beneficial moiety in controlling the oxidative-induced apoptotic events during insecticide chlorpyrifos exposure in rats [132]. It was witnessed that for chlorpyrifos treated rats, a substantial drop in the protein expression level of Bcl-2 occurred, but at the same time, a notable increase in the expression levels of Bax, cytochrome c, caspase-8, and caspase-9 was observed in the cerebrum and cerebellum, thus justifying the role of quercetin as a neuroprotective agent.

Among the isolated flavonoids from the medicinal herb *Achyrocline satureioides* Lam D.C. (Compositae) ‘marcela’ quercetin was found more effective as compared to luteolin and isoquercitrin in providing neuroprotection [133]. In hypoxia-induced injury of pheochromocytoma (PC-12) cells, quercetin was found to be effective by activating AMPK and Wnt/β- catenin signaling pathways via down-regulation of miR-122 as reported by Yan et al. [134]. Quercetin was reported to be found in the brain and other organs, including tumors induced in mice for studying the intake of *Ilex paraguariensis* A. St.-Hil [135]. They hypothesized that quercetin and chlorogenic acid present in *Ilex paraguariensis* A. St.-Hil, if taken in diet, can reduce lung cancer-related neuroinflammation. Chlorpyrifos, a pesticide, has been found to alter neuro-histoarchitecture. After treatment with quercetin, a significant improvement was observed. It was suggested that quercetin prevents chlorpyrifos-induced neurotoxicity [136]. The quercetin derivatives obtained from apple tree leaves showed a significant neuroprotective effect at low concentrations of 0.5–1.0 mg/L as compared to other phytoconstituents [137]. The results presented by Chatterjee et al. [138] revealed the neuroprotective importance of quercetin in treating dorsal root ganglion (DRG) neurons exposed to radiation-induced ER stress. The neuroprotective role of quercetin against oxidative stress-related neurodegenerative ailments in cortical brain tissue cultures from Wister rats was studied [139]. Further, the effective role of quercetin in neuron survival, Polyphenol-enriched micronutrient mixture, PMM containing quercetin was also studied for the neuroprotective potential [140]. The neuro potential of PMM was justified by explaining the inhibitory activity on CBP/p300 or stimulating activity on the AMP-activated protein kinase–sirtuin 1 pathway. *Achyranthes aspera* L. aerial parts were subjected to alcoholic extraction to examine their cerebroprotective potential [141]. The detailed study presented explained the suppression of ROS generation nitrite and TNF-α in LPS activated RAW 264.7 cell lines. Quercetin treatment reduced the increase of neurological deficit and infarction, as reported by Park et al. [142] by explaining the respective biochemical pathways and expressions. Quercetin has shown anti-hypoxic-ischemic injury (HI) in neonatal mice following the IKK-β/NF-κB pathway, as reported by Jing et al. [143]. Thus, supporting quercetin as a significant neuroprotective role in preventing brain injury. In an experimental study, administration of quercetin with different doses in 30 male Wistar rats was carried out wherein it was suggested that quercetin could be an alternative for the treatment of spinal cord injury [144]. Kumar et al. [145] reported improved neuronal survival after exposure to HgCl_2_ using quercetin and caffeine in hippocampal neurons. The term ‘disease-modifying drugs’ was referred to as these phytochemicals as these can serve as future drugs for neurological disorders (e.g., Alzheimer’s disease). The alcoholic and hexane extract of pumpkin flowers (*Cucurbita maxima* Duchesne ex Poir) were investigated for neuroprotective potential. The chromatographic results revealed that quercetin was found in high concentrations. The cholinesterases (acetylcholinesterase and butyrylcholinesterase), monoamine oxidase (MAO), and Na^+^/K^+^ ATPase activities were carried out to establish the neuroprotective activity of extracts [146].

Various phytochemicals, including quercetin derivative, were isolated from natural tea *Armeniaca sibirica* L., and their neuroprotection role was studied by Zhang et al. [147]. They hypothesized that *Armeniaca sibirica* L. has promising potential use in the food industry and addresses neuroprotective issues. Diabetes is associated with brain damage in mice as stated by Zhu et al. [148] the use of quercetin at high doses increased the levels of reduced glutathione and activity of superoxide dismutase and thus decreased the levels of advanced glycation end-products. The improvement in diabetic encephalopathy using quercetin was established by citing the decrease in protein glycation, oxidative stress, and inflammation through the upregulation of Glo-1 [148]. Quercetin at the dosage of 50 mg/kg body weight was reported to help to decrease the symptoms of Alzheimer’s disease [149]. The microscopic analysis revealed that quercetin suppressed H_2_O_2_- induced changes in cell membrane elasticity and morphological properties, leading to the hypothesis of the neuroprotective role of quercetin [150]. The iron nanoparticles loaded with quercetin were evaluated for crossing the brain barrier in Wistar rats. After an oral dosage of 100 mg/kg body weight of quercetin, the plasma and brain tissue were found to contain a higher amount of iron nanoparticles as compared to neat quercetin. This availability has suggested that these loaded nanoparticles can be used as neuroprotective agents [151]. The alcoholic extract of *Colebrookea oppositifolia* Smith. was found to be rich in quercetin as detected via LC-MS results by Viswanatha et al. [152]. The in vivo experiment was carried out in Wistar rats and found to exhibit a cerebroprotective effect. The inhibition of ROS, nitrite, and TNF-α in LPS-stimulated RAW 264.7 cell lines has supported its neuroprotective effect. The reduction of size in quercetin to the nanometric range will increase its bioavailability at the targeted site, as stated by Ghaffari et al. [153]. The 6-hydroxydopamine (6-OHDA) induced Parkinson-like model in male rats was studied by them, who reported that treatment with nanoquercetin prevented the disruption of memory while increasing antioxidant enzymatic activities such as SOD and catalase decreased the Malondialdehyde (MDA) level in the hippocampal area of the brain, thus supporting their provided hypothesis of using quercetin as an anti-Parkinson agent in rats [153]. Similarly, the combination of quercetin with bio-enhancer piperine has been reported [154] to counter 6-OHDA-induced Parkinson’s diseases in rats. The quercetin was effective against radiation-induced brain injury in Wistar rats [155]. The antioxidant and histopathological results revealed that quercetin is favorable as a neuroprotective agent. The quercetin was found active against levodopa-induced toxicity in neuroblastoma (SH-SY5Y) cells of olive biophenols, and based on its enzymatic reaction, it has been hypothesized as a promising natural inhibitor against the histone deacetylases (HDAC) enzyme by Omar et al. [156]. The traumatic brain injury (TBI) in rats were treated with quercetin using Neurological Severity Scoring, immune histochemical, and Western blot analyses. The use of quercetin exhibited significant inhibition in extracellular signal-regulated kinase 1/2 phosphorylation and activated Akt serine/threonine-protein kinase phosphorylation, thus proving its neuroprotective potential [157]. The pesticide-induced oxidative stress in the brain of rats was treated with quercetin. The reduction in oxidative stress and increases expression of paraoxanase2 enzyme (PON2) in cultured neural cells led to neuroprotection against oxidative exposure. Thereby, it proved its role as a neuroprotective agent [158]. Quercetin and biapigenin into poly (Ɛ-caprolactone) (PCL) nanoparticles were studied for their neuroprotective behavior by Oliveira et al. [159]. The focal cerebral ischemia was induced in male Sprague Dawley rats, and after treatment with quercetin neurological function test, brain edema measurement, and 2,3,5-triphenyl tetrazolium chloride staining were carried out to establish its effectiveness as a neuroprotective agent. It was hypothesized that quercetin was successful in preventing the middle cerebral artery occlusion induced activation of apoptotic pathways affecting caspase-3 and poly ADP-ribose polymerase expression, thereby establishes its neuroprotective candidature [160]. Quercetin has been found extremely beneficial for nerve regeneration in the Sprague Dawley rat model of sciatic nerve crush injury using histopathological, morphometric, and biochemical methods as reported by Türedi et al. [161]. Domoic acid (DA), a well-known neurotoxic agent, damages the hippocampal area in the brain. Wang et al. [162] studied the effect of quercetin on DA-induced neurotoxicity in mice. To their findings, a reduction in mitochondrial dysfunction via increase of AMPK activity, coupled with an increase in Nrf2 pathway mediated oxidative defense has been cited as a probable reason to hypothesize quercetin as a neuroprotective agent. Traumatic brain injury (TBI) and the potential role of the PGC-1α pathway neuroprotection using quercetin were studied in Wild-type mice by Li et al. [163]. Quercetin treatment can reduce brain injury in TBI model mice by increasing the activities of mitochondrial biogenesis through the mediation of the PGC-1α pathway. The neuroprotective behavior of quercetin in rats can be attributed to the inducing effects on the expression levels of the brain-derived neurotrophic factor mRNA [164].

The membrane permeability is a concerning issue while delivering quercetin at the injured brain site during ‘Cerebral ischemia–reperfusion’, which results due to the generation of reactive oxygen species. Thus, quercetin was loaded on polymeric nanocapsules and directed towards mitochondria and was able to higher brain uptake, thereby controlling both generations of ROS as well as the specific delivery of quercetin in the brain [165]. The effect of quercetin on hippocampal neurons after H_2_O_2_ and Aβ-induced neurotoxicity was treated with quercetin. It was hypothesized by Godoy et al. [166] that quercetin can be considered as extraordinary means for justifying and explaining molecular mechanisms associated with Aβ neurotoxicity. The extract from *Bougainvillea glabra* Choisy leaves extract rich in quercetin was considered effective in preventing neurological disorders. The study was supported based on the results of improved locomotor performance and decreased AChE activity, ROS production, and lipid peroxidation [167]. Similarly, extracts from *Bacopa monnieri* were found active against induced oxidative damage in neuroblastoma IMR32 cells. The extract was found to contain quercetin and other phytochemicals; however, the individual role of phytochemicals was not elaborated [168]. The vegetable extracts of *Vernonia amygdalina* Delile and *Struchium sparganophora* L. Kuntze. were found to show neuroprotective properties. This activity was attributed to stimulation of Na^+^/K^+^ ATPase activity, inhibition of 5′-nucleotidase, acetylcholinesterase, butyrylcholinesterase, and monoamine oxidase activities, as well as Fe^2+^, induced oxidative stress [169]. Quercetin was found active against D-galactose-induced neurotoxicity in mice, as reported by Dong et al. [170]. The presented results stated that quercetin was able to prevent changes in the neuronal cell morphology with Nrf2, HO-1, and SOD expression along with hippocampus. In vitro studies reported by Aluani et al. [171], chitosan-based quercetin nanoparticles exhibited neuroprotective activity. The quercetin in zein nanoparticles was observed to be a potential candidate for oral treatment of Alzheimer’s disease [172]. Gupta et al. [173] evaluated the protective effect of quercetin in cadmium-induced cognitive deficits in rats. They hypothesized that cadmium-induced neurotoxicity could be modulated by quercetin via molecular targets involved in brain cholinergic signaling. The continuous treatment of quercetin for 30 days in mice improved the learning and memory loss induced by amyloid β (Aβ)-peptide exposure. Thus, it was hypothesized that quercetin could be used as a treatment of neurological disorders [174]. Alzheimer’s and Parkinson’s disease, both neurological disorders, were hypothesized to be treated by extracts of edible plants *Veronica teucrium* L. and *Veronica jacquinii* Baumg methanol extracts containing quercetin and its derivatives as reported by Živković et al. [175]. The reduction in enzymes such as acetylcholinesterase (AChE) and tyrosinase (TYR) concentration after extract treatment led to such a hypothesis. Quercetin has been found in sea buckthorn (*Hippophae rhamnoides* L.) leaves, along with other phytochemicals. The ethyl acetate fraction containing these phytochemicals has been found active and increased the viability and membrane integrity of neuronal PC-12 cells [176]. Quercetin was found to increase the cell viability in PC-12 cells, along with a reduction in lactate dehydrogenase (LDH) after exposure to hydrogen peroxide, further reducing the apoptosis of PC-12 cells and hippocampal neurons were reported by Bao et al. [177] thus hypothesizing its role as new potential anti-neurodegenerative agent, particularly towards the neurodegeneration due to oxidative stress. An in vivo study in rats for 1-Methy-4-phenyl-1,2,3,6-tetrahydropyridine (MPTP)-induced neurotoxicity by quercetin in combination with piperine at different doses was studied by Singh et al. [178]. The reported results revealed that this combination was quite active in terms of antioxidant and anti-inflammatory properties as the activity of quercetin was further boosted by the presence of piperine; furthermore, quercetin, along with piperine, showed resilient neuroprotective effects once MPTP neurotoxicity was induced in Wistar rats [178]. The ethyl acetate fractionated alcoholic extract of *Allium cepa* Lam. containing quercetin was studied for diabetic neuropathy in Sprague Dawley rats. The significant neuroprotective effect was observed after 14 days of quercetin administration against streptozotocin-induced diabetic neuropathy [179]. Alligator weed (*Alternanthera philoxeroides* (Mart.) Griseb.) extracts were also found rich in quercetin along with other phytochemicals as reported by Khamphukdee et al. [180] and their neuroprotective role was also stated by reporting anti-anxiety property in mice through estrogenic activity. Similarly, *Carpobrotus edulis* L. extracts containing quercetin were found to inhibit acetylcholinesterase (AChE) and butyrylcholinesterase (BuChE), which were able to reduce hydrogen peroxide (H_2_O_2_)-induced injury in the human dopaminergic cell line SH-SY5Y as reported by Rocha et al. [181]. In a similar study, flowers of *Reynoutria sachalinensis* F. (Schmidt.) Nakai. contained quercetin neuroprotective activity as revealed by Eom et al. [182]. Ay et al. [183], in their detailed study, explained the role of quercetin and hypothesized that the cell survival signaling axis was activated by PKD1-Akt using quercetin, which can be a potential candidate for clinical use in treating PD. Among many phytochemicals obtained from *Potentilla parvifolia* Fisch. (Rosaceae), quercetin exhibited better neuroprotective effects as reflected by Western blot and morphology analysis [184]. Vegetables such as cabbage (*Brassica oleracea* var. capitata L.) and cucumber (*Cucumis sativus* L.) were subjected to aqueous extraction, and in vitro enzymatic activity of acetylcholinesterase, butyryl-cholinesterase, and monoamine oxidase was inhibited. It was hypothesized that these vegetables could be used to treat neurodegeneration and were stated as probable sources of functional foods and nutraceuticals for the management of Alzheimer’s diseases [185]. *Gynostemma laxum* Wall. Cogn. containing quercetin has shown neuroprotective activities and was suggested a natural antioxidant supplement, but Seo et al. [186] have warned further in-depth in vivo investigation. The erythropoietin levels were studied in traumatic brain injury in a rat model, and its levels were unaltered by the use of quercetin, thus justifying its neuroprotective role [187]. Quercetin was used to inhibit acid-sensing ion channels (ASICs), which are associated with neuronal death during ischemic stroke, epileptic seizure, and nociception, as reported by Mukhopadhyay et al. [188]. With the help of computational analysis, they were able to prove the inhibitory effect of quercetin on ASICs and suggested that further in vivo studies are possibly required to show that quercetin can target ASICs. The crossing of the blood–brain barrier (BBB) along with rescuing degenerated neurons, acting on Alzheimer’s disease quercetin was loaded into colloidal formulation symbolled as RMP-7-Lf-QU-LS by Kuo and Tsao [189], where RMP-7 is a bradykinin analog, lactoferrin (Lf), quercetin (QU), and liposomes (LS). The results revealed that this colloidal formulation was effective in overcoming BBB to prevent Aβ-induced neurodegeneration and can be used to manage Alzheimer’s disease in proposing future clinical applications. Quercetin was used to check the memory impairment and deterioration of the cholinergic system in rats with an acquired or genetic disorder of having high levels of lipids in the bloodstream. The increased level of AChE activity in such rats suggested that quercetin can prevent memory impairment and change lipid metabolism [190]. In a similar study, the restoration of acetylcholinesterase activity, an increase of redox status, and lipid peroxidation inhibition in the brain of rats were controlled by the use of quercetin in manganese-induced neurotoxicity [191]. The quercetin and its metabolite quercetin-3-*O*-glucuronide were able to control neural stem cell viability as reported by Baral et al. [192] during an in vivo and in vitro study via contrary regulation of protein kinase B (Akt). They presented results wherein it was revealed that the reported mechanism suggests quercetin metabolite to possess therapeutic potential in treating neurodegenerative ailments. Quercetin was found effective against sodium fluoride-induced neurotoxicity in rats, as confirmed by Mesram et al. by evaluating biochemical, behavioral, and histopathological parameters [193]. Mehta et al. [194] found quercetin effective in improving memory dysfunction and rescued neurons from chronic unpredicted stress-mediated damage. The molecular mechanism of quercetin showing anticonvulsive behavior was studied in a mouse model by Moghbelinejad et al. [195]. The results revealed that GABAA α5 receptor, which controls the inhibitory signaling in the central nervous system, was probably responsible for this anticonvulsive behavior of quercetin, and a further mode of action was warned for further in-depth studies. *Salicornia herbacea* L. extracts possessing quercetin were found to provide a neuroprotective effect via regulating the expression of antioxidant proteins (Nrf2) facilitated introduction of antioxidant enzymes [196]. The quercetin-protected hippocampal CA1 pyramidal neurons from ischemic injury was studied by Chen et al. [197], who established their role in providing resilient neuroprotective effects against transient cerebral ischemia by increasing the expression of antioxidant enzymes. Quercetin, in combination with donepezil, enhances cognitive memory in rats by reducing AchE, β amyloid1-42 level in the rat brain [198]. The quercetin was able to protect neuronal damage in the retina of diabetic rats by improving BDNF, NGF, TrkB, synaptophysin, Akt, Bcl-2, cytochrome c, and caspase-3 expression using Western blotting techniques [199]. The Wistar rats were given quercetin supplementation for evaluating neuroprotective effects such as a decrease in neuronal and glial body areas along with a reduction in neuronal and glial density [200]. Quercetin and its nanocrystal at different concentrations were given orally to rats and increased behavioral indexes along with the anticipation of anxiogenic-like behavior in them [201]. Eggplant (*Solanum melongena* L.) extract containing quercetin was evaluated for its neuroprotective activity against 6-hydroxydopamine induced in the Parkinsonian rat model [202]. Based on these reports, quercetin used for neuroprotective use has been tremendously covered. The main mechanism is either focus on enzymatic reaction, animal models, and dose dependency. These studies can infer the strongest use of quercetin as a future drug for neurogenerative diseases. The source of quercetin is of utter importance, although that obtained from flora, including vegetables and fruits, to support a circular economy, more plant waste, particularly food waste, should be exploited as a quercetin source. Some recent studies highlighting the role of quercetin as a neuroprotective agent are depicted in Table 2.

## 3. Quercetin from Food Industry Wastes and By-Products 

Food industries, mainly post-fruits and vegetable processing, generate a massive amount of waste/byproducts, which are usually discarded as landfilling or are subjected to composting and incineration, which remains a common process of waste disposal. Managing this type of waste/byproduct is vital, especially to reduce environmental pollution. However, it is worth noting that this cheap source of raw material occurring as waste/byproducts can be a worthy source of bioactive compounds such as quercetin, exhibiting rich bioactivities. These bioactives obtained have been explored for their antioxidant, anti-microbial, anti-tumor activities, as well as have been recommended to be used as a natural coloring and a preservative agent in different industries (such as in pharmaceuticals, cosmetics, and food industries). Though a wide range of compounds has been obtained, in the present context, our focus remains on quercetin obtained from vegetal wastes. Quercetin has been obtained from apple and plum pomace [297,298,299] as well as from the peel wastes of banana, kiwi, tomato, and guava fruits [300,301,302,303]. Skin wastes of onion and garlic have been reported as a good source of polyphenolic compounds such as quercetin, which was shown to exhibit certain bioactivities [304,305,306]. Besides, quercetin has been isolated from the peels of different citrus varieties [307]. Polyphenolic compounds such as gallic, vanillic, syringic, and ferulic acids and *p*-coumaric derivatives along with flavonoids such as those of quercetin and its derivatives have been obtained from the ‘lees’ generated post ‘pisco’ production process [308]. Among the fruit seed wastes, grape seeds are reported to be rich in polyphenols, resveratrol, quercetin, and other flavonoid compounds, which are confirmed to impart cardiovascular protective effects [309]. In papaya seeds, quercetin and quercetin 3-*O*-glycoside have been isolated [310]. Furthermore, seeds of *Theobroma grandiflorum* (a tree related to cacao) are reported to be a rich source of bioactive sulfated flavonoid glycosides, quercetin, quercetin 3-*O*-D-glucuronide, and other polyphenolic compounds [311]. Quercetin and isoquercetin have been obtained from the mango seed kernel, too [312].

On another note, among various types of leaves studied, quercetin-3-*O*-(6″-feruloyl)-β-D-galactopyranoside was obtained from guava plants leaves (*Psidium guajava* L.), which demonstrated rich bioactives [313], while quercetin-3-*O*-rutinoside, quercetin-3-orobinobioside and quercetin-3-*O*-galactoside were isolated from chokeberry leaves [314,315] In the cranberry leaves (+)-catechin, procyanidin B1, (−)-epicatechin have been isolated [314]. From the quince leaves, quercetin-3-*O*-galactoside, quercetin-3-*O*-rutinoside have been obtained [314,316]. On another note, elderberry (*Sambucus nigra* L.) branch wastes have been explored to obtain quercetin and its glycoconjugate derivatives [317]. Residues obtained from broccoli-processing industries (stalks and florets) have been explored to obtain glucosinolate, polyphenols, and flavonoids such as quercetin [318]. Cauliflower wastes extracts (stems and leaves) are also reported to contain high amounts of flavonoid glycosides, mainly derived from quercetin and kaempferol [319,320]. Apart from fresh produce waste, quercetin has been reported in other wastes such as sugarcane (*Saccharum officinarum* L.), bagasse [321,322], acerola (*Malpighia emarginata* DC.), flour bagasse [323], stalks of *Euonymus alatus* (Thunb.) Sieb. [324], and peanut shells [325]. With the available information in the scientific databases, it is quite evident that food industrial wastes/byproducts (mainly those of fruits and vegetables based) hold high scope for obtaining quercetin. The food waste/byproducts containing quercetin by the amount reported in recent years are summarized in Table 3.

## 4. Conclusions

The tremendous effort by researchers to bring attention to the importance of quercetin as a cardioprotective and neuroprotective agent has led to a new milestone in discovering it as a potential therapeutical candidate. The sustainable resources from plant sources, in particular, vegetables, have added value to this research. A paradigm shift is needed to utilize food waste or food byproducts as a continued sustainable resource of this secondary metabolite. The main challenge in tabulating quercetin as a cardioprotective and neuroprotective agent is the diversion of demand from fresh resources to non-utilized food waste. However, other challenges, such as the specific targeted delivery of quercetin and its understanding of the cellular metabolism of quercetin derivatives, need a more interdisciplinary approach. The use of nano-particulate quercetin can be carried out in the future based on the sources of origin, which in turn can increase the bioavailability of quercetin at the drug-targeted site for cardiovascular and neurological diseases. The cellular mechanism, targeted delivery, and above all effectiveness, of quercetin in treating cardioprotective and neuroprotective diseases has been remarkably covered by researchers; however, the quercetin sources from fresh fruits and vegetables and synthetic methods are needed to be diverted to food wastes or byproducts. Further research is also required to identify the purity of quercetin, its safety efficacy, and meet the standard regulations of governing bodies (e.g., EU legislation). Thus, this review warrants further extensive research activities to be undertaken to obtain quercetin from underexplored plant produce (e.g., vegetables and fruits) as well as food-industry processing wastes and byproducts, mainly originating from fruits and vegetables.

## Figures and Tables

**Table 1:** Some recent reports on the role of quercetin as a cardioprotective agent.

Role	Mode	Reference
Translocation of NFκB, and expression of TGF-β1, CTGF, and BNP	In vivo	[43]
Cardioprotective effects restoring plasma thyroid hormone levels and attenuating oxidative stress in the heart	In vivo	[44]
Inhibition of JNK and p38 mitogen-activated protein kinase signaling pathways	In vivo	[45]
Inhibits angiotensin-converting enzyme activity, improves vascular relaxation, and decreases oxidative stress and gene expression	In vitro, in vivo	[46]
Post-ischemic recovery of left ventricular developed pressure, as well as recovery of markers of contraction and relaxation, respectively	In vivo	[47]
Cardioprotective	In vivo	[48]
Myocardial infarction Release of creatine kinase-MB (CK-MB), lactate dehydrogenase (LDH) level in coronary effluent and reduced myocardial infarct size	In vivo	[49]
Myocardial ischemia-reperfusion injury via suppressing the NF-κB pathway	In vivo and in vitro	[50]
Protein disulfide isomerase (PDI) inhibition for cardiovascular benefits	In vitro	[51]
Protects cardiomyocytes against oxidative toxicity and the regulation of stress-sensitive protein kinase cascades and transcription factors.	In vitro	[52]
Lowers ABP in patients with hypertension	In vivo	[53]
Cardioprotective control	In vitro	[54]
Endothelial function and reducing inflammation, vascular function, and cardiometabolic health	In vivo	[55,56]
Improve cyclosporine-induced cardiotoxicity, as it has antioxidant and anti-inflammatory enzyme activities	In vivo	[57]
Atherogenic and cardioprotective indices	In vivo	[58]
Reduction in cardiac and renal markers of oxidative stress	In vivo	[59]
Antichronic doxorubicin cardiotoxicity via antioxidant and anti-inflammatory properties	In vivo	[60]
Antioxidative cardiotoxicity and dyslipidemia	In vivo	[61]
Cardiac weight index and myocardial enzyme activity Antioxidative stress, inhibition of the renin–angiotensin–aldosterone system	In vivo	[62]
Ischemia/reperfusion injury in cardiomyocytes	In vitro	[63]
Anti-doxorubicin-induced cardiomyopathy in H9c2 cell; myocardial ischemia/reperfusion injury in rats through the PI3K/Akt pathway	In vitro	[64,65]
Induce activation of AMPK and eNOS in human aortic endothelial cells	In vivo	[66]
Cardioprotective effect of GSK-3b inhibitors	In vivo	[67]
Reduction of the serum CK-MB, LDH, and SGPT level enzymes	In vivo	[68]
Protects rat hearts from oxidative stress by its antioxidant potential	In vivo	[69]
Decrease in doxorubicin-induced cytotoxicity and promoting the cell repair system in cardiomyocyte H9C2 cells	In vitro	[70]

**Table 2:** Some recent reports on the role of quercetin as a neuroprotective agent.

Role	Mode	Reference
Antioxidant and AChE inhibitory activity	In vitro and in vivo	[203]
To prevent the increase in AChE activity in the brain, improve the memory and anxiety-like behavior	In vivo	[204]
Proteasome activities	In vitro	[205]
Against oxidation-induced neuronal necrotic-such as cell death	In vitro	[206]
Modulation of neuroinflammation and the cholinergic system	In vivo	[207]
Neuronal autophagy and brain injury model by activation of PI3K/Akt signaling pathway	In vivo	[208]
Endoplasmic reticulum stress and neuronal cells	In vitro	[209]
Reduced oxidative/nitrative damage to DNA, lipids, and proteins of neuroblastoma cell line (SH-SY5Y) cell	In vitro	[210]
Improves ischemia/reperfusion-induced cognitive deficits. Inhibition of ASK1/JNK3/caspase-3 Akt signaling pathway	In vitro	[211]
Prevention of brain damage by acrylamide	In vivo	[212]
Inhibition of μ-calpain protein in hypoxia-induced neuronal injury	In vitro	[213]
Autophagy-modulating, Parkinson’s diseases	In vivo	[214]
Deprivation and restoration of oxygen/glucose, increased the expression of Nrf2	In vitro	[215]
Neuronal death prevention	In vivo	[216]
Anti-convulsant	In vivo	[217]
Inhibition of monoamine oxidase (MAO), AChE, and BChE activities	In vitro	[218]
Antioxidative insult	In vivo	[219]
Cerebroprotective action	In vivo	[220]
Prevention of okadaic-acid-induced injury by MAPK and PI3K/Akt/GSK3β signaling pathways	In vitro	[221]
Reduction of oxaliplatin-induced oxidative stress in brain	In vitro and in vivo	[222]
Reduction of immunoreactivity of degenerating neurons	In vivo	[223]
Parkinson’s disease	In vivo	[224]
Apoptosis on neural cells via PI3K/Akt signal pathway	In vitro	[225]
Cerebrovascular disorders	In vivo	[226]
Alzheimer’s disease (AD) prevention	In vivo	[227]
Defense of oxidative Stress via PKC- ϵ inactivation/ERK1/2 activation	In vivo	[228]
Neuropathic pain reliever	In vitro	[229]
Inhibiting oxidative stress and inflammation in brain injury	In vivo	[230]
Hypoxic–ischemic brain injury	In vivo	[231]
Alzheimer’s disease prevention	In vitro	[232]
Anti-neuroinflammatory	In vitro	[233]
Enhanced neuronal mitochondrial performance	In vitro	[234]
Brain therapy, hypoxia	In vivo	[235]
Anti-inflammatory, antioxidant, and anti-acetylcholinesterase activities in	In vitro	[236]
Reduction in oxidative-stress-mediated neurodegeneration	In vivo	[237]
Prevention of Parkinson’s disease by gene expression	In vitro	[238]
Anti-brain ischemic/reperfusion injury using Akt pathway	In vivo	[239]
In neuron survival	In vitro	[240]
Cognitive function	In vivo	[241]
Antioxidative stress, neuronal damage,	In vivo	[242]
Protection of human brain cells	In vitro	[243]
Spatial memory dysfunctions improvement	In vivo	[244]
Protection of cognitive and emotional functions	In vivo	[245]
Reduction of cell apoptosis of oxidant-stressed neuroblastoma (SK-N-MC) cells	In vitro	[246]
Protects the weakening of memory and anxiogenic behavior	In vitro	[247]
Locomotor activities, neurotransmission	In vivo	[248]
Spinal cord injury treatment	In vitro	[249]
Perinatal cerebral hypoxia–ischemia	In vivo	[250]
Protection from oxidative stress and brain edema	In vivo	[251]
Brain protection	In vivo and in vitro	[252]
Retinal neuroprotection	In vivo	[253]
Brain injury treatment	In vivo	[254]
Neurolemmocytes damage prevention	In vivo	[255]
Protection of PC12 neural cells	In vitro	[256]
Multiple therapeutic molecular targets of Alzheimer diseases	In vitro	[257]
Reduction of neuroinflammatory response, antidepressant	In vivo	[258]
Prevention of hippocampal nerve damage, improved memory function	In vivo	[259]
Neuron density	In vivo	[260]
Prevention of chemical hypoxia	In vitro	[261]
Inhibition of glutamate release	In vitro	[262]
Anxiolytic effects	In vivo	[263]
Ectoenzymes and acetylcholinesterase activities	In vivo	[264]
Increases levels of mitochondrial enzyme (PON2) in brain cells	In vivo	[265]
Nerve protection via Nrf-2/HO-1 activation and NF-κB inhibition	In vivo	[266]
Catalepsy normalization, improvement of neurochemical parameters	In vivo	[267]
Suppression of cellular acetylcholinesterase (AChE), protection against oxidative stress	In vitro	[268]
Preventive medicine for polychlorinated biphenyls (PCBs)-induced neurotoxicity	In vivo	[269]
Cerebral ischemia–reperfusion injury treatment	In vivo	[270]
Protection against induced neurobehavioral impairments	In vivo	[271]
Neuroprotection in mitochondrial neurotoxin-induced Parkinson diseases	In vivo	[272]
Neurovascular coupling protection, decrease in neurovascular oxidation	In vivo	[273]
Neuronal protection	In vivo	[274]
Neuroprotection against brain oxidative stress	In vivo	[275]
Against neuron death	In vivo	[276]
Against neurotoxic venoms	In vitro	[277]
Neurodegeneration protection via production of ROS scavenging	In vivo	[278]
Neuroprotection in ypoxic–ischemic brain injury	In vivo	[279]
Neuroprotection in duodenum enteric nervous system	In vivo	[280]
Alcoholic neuropathy protection	In vivo	[281]
Protection in cerebral ischemia through activation of BDNF-TrkB-PI3K/Akt signaling pathway	In vivo	[282]
Prevention of oxidative stress in brain	In vivo	[283]
Reversal of hypobaric hypoxia, neuroprotective response stimulant	In vivo	[284]
Diabetic neuropathy prevention	In vivo	[285]
Prevention of oxidative damage by induced neurotoxicity	In vitro	[286]
Prevention of cerebral ischemia-induced oxidative stress	In vivo	[287]
Neuroinflammation prevention	In vitro	[288]
Cerebral ischemia protection	In vitro	[289]
Protection Oxidative injury P19 neurons	In vitro	[290]
Lutamate-induced neurotoxicity protection in HT22 cells	In vitro	[291]
Neuron cell protection	In vitro	[292]
Oxidative stress	In vivo	[293]
Decreases the neuronal damage and scavenged free radicals	In vivo	[294]
Protection for cerebral ischemic conditions	In vivo	[295]
Neurodegeneration protection	In vivo	[296]

**Table 3:** Quercetin in food industrial wastes and byproducts.

Waste/By-Products	Quantity (mg/g) *	Reference
Tomato peels	9.97 ± 0.27	[326]
Berry peel	0.0001 ± 0.00	[327]
Lotus byproducts	(Only detected not quantified)	[328]
Coppery onion outer dry layers	52.84 ± 0.12	[329]
Red grape pomace	0.05 ± 0.00	[330]
Cacao beans pod husk	0.6018 ± 0.0112	[331]
Grape pomace	0.03189 ± 0.00277	[332]
Grape pomace	0.24923 ± 0.00114	[333]
Onion waste	(A case study of industrial scale, output yield is in Kgs)	[334]
Black currant residue with quercetin glycoside (based on the place of cultivation)	34.6 ± 5.7	[335]

* The units presented are converted to (mg/g) as compared to different units reported in the references.

## Data Availability

Not applicable.
